# Aqueous Tuber Extracts of *Tylosema fassoglense* (Kotschy ex Schweinf.) Torre and Hillc. (Fabaceae). Possess Significant *In-Vivo* Antidiarrheal Activity and *Ex-Vivo* Spasmolytic Effect Possibly Mediated by Modulation of Nitrous Oxide System, Voltage-Gated Calcium Channels, and Muscarinic Receptors

**DOI:** 10.3389/fphar.2021.636879

**Published:** 2021-03-16

**Authors:** Washika Amos Mapesa, Mwangi Peter Waweru, Frederick Bukachi, Kayaja David Wafula

**Affiliations:** Department of Medical Physiology, School of Medicine, University of Nairobi, Nairobi, Kenya

**Keywords:** spasmolytic, nitrous oxide, muscarinic, antidiarrheal, traditional medicinal herbs

## Abstract

*Tylosema fassoglense* (TFG) is used as an antidiarrheal traditional medicine in Western Kenya. This study aimed to investigate the antidiarrheal activity of its aqueous extracts *in-vivo* and the putative mechanism (s) of action *ex-vivo* using Sprague-Dawley rats and New Zealand white rabbits respectively. The *in-vivo* antidiarrheal effects of the extract were evaluated in castor oil-induced diarrhea, the castor oil-induced enteropooling, and phenol red gastric motility tests. On the other hand, isolated rabbit’s jejunal segments were used to evaluate the spasmolytic effect of TFG on spontaneous contraction, in acetylcholine-induced contraction, in presence of 80mMK^+^, calcium chloride-induced contraction as well as in presence of the following antagonists: naloxone, methylene blue, L-NAME, prazosin, and propranolol in the *ex-vivo* studies. The data were express as Mean ± S.E.M and analyzed by one-way ANOVA and Tukey’s post hoc test in cases of significance which was set at *p* < 0.05. The extract was phytochemically characterized using Liquid chromatography Mass spectroscopy (LC-MS).The extract possessed significant inhibitory effect in the *in-vivo* experiments. The extract exhibited significant spasmolytic effect on both spontaneous contraction and in jejunal segment pre-contracted acetylcholine as well as in presence of 80mMK^+^ solution. It also attenuated the spasmogenic effect of various concentration of calcium chloride. The extract’s spasmolytic effect was, however, significantly attenuated in presence of several antagonists (methylene blue and L-NAME) but the adrenergic blockers (prazosin and propranolol) had no significant effect in the *ex-vivo studies.* LC-MS identified thirty compounds where Proathocyanidin (11.54%), Syringic acid (7.30%), and 4-Hydroxybenzoic acid (6.19%) had the highest percentage abundance. In conclusion, the results obtained in this study partially validate the traditional uses of the tubers of this plant species as an antidiarrheal. These antidiarrheal effects are probably mediated via modulation of nitrous oxide pathway, voltage gated calcium channels, and muscarinic receptors.

## Introduction

Diarrhea is a gastrointestinal disorder characterized by an increase in frequency of bowel movement of at least three times or more per day (Levine et al., 2017), increased liquidity of stool (Shane et al., 2017), and an increase in amount of stool of at least 200 g/day (Levine et al., 2017). Diarrheal diseases lower the quality of life, prolong hospitalization stays, raise healthcare costs and are the leading cause of death in children aged below five years (DuPont, 1995; Kotloff et al., 2017). These health impacts are attributed to the link between diarrhea and pre-renal acute kidney injury (Seifter and Chang, 2017) , malnutrition in patients (Guerrant et al., 1992) , and deterioration of health especially when comorbid with chronic diseases (World Health Organization, 2019). Indeed, diarrheal diseases account for one in nine deaths of children under the age of 5 (Centers for Disease Control and Prevention, 2015). The death rate is eleven-fold higher for children with comorbid conditions such as HIV (Centers for Disease Control and Prevention, 2015). Developing countries inordinately bear a huge global burden of diarrheal diseases (Merson, 1981; Kotloff et al., 2017).

Diarrhea is classified into five interrelated categories: secretory (Harrell and Cheng, 2018), malabsorptive (Hammer et al., 1990; Walters and Pattni, 2010), osmotic (Binder, 2010), motility, and congenital (Na^+^ or Cl/HCO^−^_3_) disorder diarrhea (Kere et al., 1999; Janecke et al., 2016). Secretory diarrhea is caused by toxins of infectious agents such as *Vibrio cholerae* which possess inhibitory effect on GTPase enzyme. As such, there is continuous activation of cAMP-adenylyl cyclase pathway, affecting gating of cystic fibrotic transmembrane regulator (CFTR) ion channel leading to hypersecretion of chloride ions into gut’s lumen and consequently water via osmosis (Harrell and Cheng, 2018). Digestive enzymes deficiency, inflammatory bowel diseases or absence of certain membrane transporters result in malabsorptive diarrhea (Surawicz, 2010). Lactase deficiency, for instance, cause the transition of the undigested lactose to the colon where microbiota convert it anaerobically into osmotically active short fatty acid anions (SCFAs) that mediate osmotic diarrhea (Binder, 2010). Similarly, unabsorbed bile salts due to the absence of apical bile salt transporters (ABST) as seen in terminal ileum resection, cause diarrhea via intestinal membrane irritation and increased mucus production (Barkun et al., 2013). Motility disorder diarrhea is mediated via neurohumoral effect where elevation of neurotransmitters or hormones such as secretin produced by tumors of enterochromaffin cells (carcinoid syndrome), shorten intestinal transit time via serotoninergic signaling (Ohevon der Ohe et al., 1993). The absence of brush boarder sodium-proton exchanger isoform (NHE3) in congenital Na^+^ disorder, causes sodium and accompanying copious fluid loss that manifest with hyponatremia (Surawicz, 2010).

Current treatment modalities of diarrhea aim at restoring fluid volume and electrolyte balance using various approaches i.e. the use of antibiotics in bacterial infectious diarrhea, bile acid sequestrant for patients with bile salts malabsorption (Porges, 1958), fecal microbiota transplant for patients with gut microbiota dysbiosis (Niccum et al., 2018) , and rotavirus vaccines for children under the age of 5 years who are susceptible to rotavirus infection (Dennehy, 2008). The use of these approaches is limited by numerous adverse effect and financial constraints. As such, there has been an increase in adoption of alternative medicine approaches in the management of diarrheal diseases (Patel et al., 2013). Some of the antidiarrheal herbal drugs in use include but not limited to *Idigofera spicata* Forssk(Fabaceae) (Awe et al., 2011), *Pyrenacantha staudtii* (Engl.) *Engl.* (Icacinaceae) (Birru et al., 2016), and *Alpinia oxyphylla Miq.* (Zingiberaceae) (Wang et al., 2015). The increase in the adoption of herbal medicine is attributed to their availability, affordability, efficacy, and reduced side effects (Patel et al., 2013). The tubers of *Tylosema fassoglense* (Kotschy ex Schweinf.) Torre and Hill (Fabaceae.) (http://www.theplantlist.org/) is a traditional antidiarrheal herbal remedy in Western Kenya (Maundu et al., 1999).

*Tylosema fassoglense* belongs to the family Fabaceae (Tropical Plants Database, 2018) with three other closely related species from the genus Tylosema which includes *Tylosema humifusa* (Pic.Serm. and Roti Mich.) Brenan, *Tylosema esculentum* (Burch.) A. Schreib, and *Tylosema argentea* (Chiov.) Brenan. (Chingwaru et al., 2015). *Tylosema esculentum* and *Tylosema fassoglense* are widely used in traditional medicine to manage various ailments (Quattrocchi, 2016). *Tylosema fassoglense* is used for the management of postpartum uterine healing, impotence, and hypertensive diseases (Quattrocchi, 2016). *Tylosema esculentum* is used among the Batswana community in South Africa in treatment of asthmatic attacks and as a protein source from its morana beans while the tubers, just like *T. fassoglense* in Western Kenya, are used in the treatment of diarrhea in oral drink preparation (Quattrocchi, 2016). *Tylosema fassoglense* contains high levels of tyrosine, lysine and proline although the levels of methionine and cysteine are relatively low (Dubois et al., 1995). The defatted meal of *Tylosema fassoglense* has considerable amount of phytates and trypsin inhibitor (Dubois et al., 1995). The ethanol extract of *Tylosema fassoglense* have shown to possess antimicrobial activity (Adongo et al., 2012).

This study aimed to investigate the antidiarrheal activity of its aqueous extract *in-vivo* and the putative mechanism (s) of action *ex-vivo* using Sprague-Dawley rats and New Zealand white rabbits respectively.

## Materials and Methods

### Plant Collection and Extract Preparation

*Tylosema fassoglense* tubers were collected from Mumias sub-county, Western Kenya. The identity of collected plant material was verified by a plant taxonomist at the University of Nairobi herbarium and a voucher specimen deposited therein (AMW 2019/001). The plant tubers were chopped into small pieces and air-dried for one week then milled to fine powder. The resulting fine powder was then macerated in distilled water in 1:10 ratio (weight/volume) for 1 h with intermittent shaking. The resulting suspension was then sequentially filtered using cotton wool followed by Whatman ^TM^ filter paper. The resulting filtrate was then lyophilized to obtain a freeze-dried extract. One thousand (1000) grams of milled plant tubers yielded thirty (30) grams of freeze-dried extract i.e. a percentage yield of 3%. The obtained freeze-dried extract was placed in amber-colored sample bottles and stored in refrigerator at + 2°C.

### Study Animals

Sixty-five adult Sprague Dawley rats of both male and female aged between 12 and 14 weeks and weighing 300 ± 50 g were used in the *in-vivo* studies. Fifteen New Zealand White Rabbits both male and female weighing 1.75 ± 0.25 kg were used in the *ex-vivo* studies. The animals were kept in the animal house within the Department of Medical Physiology, University of Nairobi. The temperature and humidity within the animal house were maintained at 22–25 °C and 60% respectively. A 12 h light: dark cycle was maintained within animal house. The animals were allowed *ad libitum* access to water and standard animal chow (UNGA Farm Care Ltd.). The animals were acclimatized for two weeks before the commencement of the experiments. The procedures used in this study are similar to those used in Li et al., (2018).

### Castor Oil-Induced Diarrhea

The antidiarrheal activity of TFG extract was investigated according to the method described by Awouters et al*.* (1978). Briefly, twenty-five Sprague Dawley rats were randomized into negative control (normal saline), positive control (5 mg/kg Loperamide), low dose test (200 mg/kg), medium dose test (400 mg/kg), and high dose test (800 mg/kg) groups (*n* = 5, per group). The experimental TFG doses were based on findings of pilot studies which indicated that doses less than 200 mg/kg of TFG had no significant effect. The rats were fasted for 24 h but with *ad libitum* access to water before the experiment. One ml of castor oil (ARANDI Castor Seed Kenya Ltd.) was administered 1 h after administration of the respective treatments by oral gavage. The animal cages were observed for 4 h for presence of diarrheal feces. The mass of diarrheal feces was determined by subtracting the weight of the filter paper from the final weight of the paper in the individual cages and recorded. The count of diarrheic stools excreted by each experimental animals was also recorded. The data were then expressed as the percentage of negative control (Awouters et al., 1978). The doses of TFG with the greatest antidiarrheal effects were used in the subsequent *in-vivo* studies.

### Castor Oil-Induced Enteropooling Test

The effect of TFG on castor oil-induced enteropooling test was evaluated using the method described by Degu et al. (2016). Briefly, twenty Sprague Dawley rats were randomized into the negative control (normal saline), medium dose test (400 mg/kg TFG), high dose test (800 mg/kg TFG), and positive control (Loperamide, 5 mg/kg) experimental groups. The rats were given 1 ml of castor oil by oral gavage 1 h after the respective treatments in each group following a 24 h starvation period but with *ad libitum* access to water. The rats were sacrificed by cervical dislocation 30 min after the administration of castor oil. An abdominal incision was made in each rat and the luminal content of the respective small intestines from the pylorus to the ileocecal junction weighed and recorded.

### Phenol Red Meal Test

The effect of TFG extract on gastrointestinal motility was investigated using the phenol red method described by Qu et al. (2014). Briefly, twenty Sprague Dawley rats were randomized into the negative control (normal saline), medium dose test (400 mg/kg TFG extract), high dose test (800 mg/kg TFG extract), and positive control (Loperamide 5 mg/kg) groups. The respective treatments were administered to the experimental animals through oral gavage after they had been starved for 24 h but with *ad libitum* access to water.

Neostigmine (0.05 mg/kg, i.p) was administered to each experimental animal 30 min after administration of the respective treatment. Phenol red meal (0.5 mg of phenol red dye per ml of 1.5% CMC) was then administered by oral gavage to each rat at a dose of (10 ml/kg) 20 min after administration of neostigmine. The experimental animals were then sacrificed by stunning 20 min after phenol red meal administration. An abdominal incision was made in each animal and the stomach and the small intestine isolated.

The amount of phenol red dye retained in the stomach was assayed and determined by spectrophotometric method Qu et al. (2014). Briefly, stomachs belonging to respective animals were each homogenized in 25 mls of 0.1 M sodium hydroxide for 30 s and the resulting homogenate allowed to settle for 1 h. One ml of trichloroacetic acid (33% w/v) was then added to 8 ml of supernatant fluid to deprotenize the supernatant which was then centrifuged for 10 min at 3200 g. One ml of 2 M sodium hydroxide was then added to 4 ml of the centrifuged supernatant and the absorbency of sample at a wavelength of 560 nm determined and recorded. The absorbency of five non-treated experimental animals given phenol red meal and sacrificed immediately served as the standard reference of a stomach with 100% phenol red dye. The gastric emptying rate was determined using the formula:

$\text{Gastric Emptying Rate}=\left[1-\left(\frac{\text{Absorbance of Test Sample at }560\text{ after }20\text{ minutes}}{\text{Absorbance of standard reference at }560\;}\right)\right]\times 100$

The distance covered by phenol red meal from pylorus was measured and expressed as percentage of total length of small intestine to find the peristaltic index.

### *Ex-vivo* Experiments Using Isolated Rabbit’s Jejunum

Fifteen New Zealand white rabbits were starved for 24 h but with *ad libitum* access to water prior to the experiment. The rabbits were euthanized by cervical dislocation and an abdominal incision performed after which 10 cm jejunal segments were isolated. The jejunum segments were dipped in beaker containing Tyrode’s solution (8 g/L NaCl, 0.2 g/L KCL, 1 g/L NaHCO3, 0.05 g/L NaHPO4, 1 g/L MaCl2, 0.2 CaCl2, and pH 7.4) which had been pre-aerated with carbogen (carbon dioxide: oxygen, 1:19) at 0°C. The jejunal strips contents were flushed out and the strips were transferred to a beaker containing Tyrode’s solution at room temperature (25°C). Two-centimeter jejunal segments were then vertically suspended in organ bath containing 40 ml of Tyrode’s solution maintained at 37°C and continuously aerated with carbogen. The proximal part of respective jejunal segment was connected to an isometric force transducer (ML500/A^TM^, AD instruments) connected to Powerlab^TM^ data acquisition system (AD Instruments).

The respective suspended jejunal segments were equilibrated for 1 h prior the evaluation of the effects of different concentrations of TFG (0.5, 1.0, 1.5, 2.0, 2.5, 3.0, and 3.5 mg/ml) on jejunal contraction. Normal spontaneous jejunal contractions were recorded for 3 min followed by a 10 min recording after addition of each concentration of TFG. The organ bath was washed out and rinsed thrice prior adding another 40 ml of fresh pre-warmed Tyrode’s solution in the organ bath while ensuring full recovery of jejunal segment before moving to the next concentration. The experiment started with lowest concentration (0.5 mg/ml) and ascended in gradations of 0.5 mg/ml to a maximum of 3.5 mg/ml of TFG. The respective contraction forces (μv) of each treatment were expressed as percentage of control.

### Mechanism of Action Experiments

The effect of various concentrations (0.5,1.0,1.5, 2.5 and 3.5 mg/ml) of TFG extract on jejunal spontaneous contraction were evaluated in presence 10^−4^ M of standard receptor blockers (prazosin- α1-adrenergic blocker, propranolol a non-selective β- adrenergic blocker, and naloxone-non selective opioid blocker) and enzyme inhibitors: L-NAME- (nitric oxide synthase inhibitor), and methylene blue (guanylyl cyclase inhibitor). The spasmogenic effect of acetylcholine and a Tyrode’s solution enriched with 80 mM potassium chloride were also evaluated in presence of various concentration of TFG. In addition, the effect of calcium chloride solutions of various concentration (0.00003M, 0.0003M, 0.003M, 0.03M) were evaluated in the presence and absence of various concentration of TFG (0.5 mg/ml, 2.5 mg/ml and 3.5 mg/ml) as well as verapamil (0.025 mM). A modified Tyrode’s solution was used in these experiments i.e. KCl concentration was raised from a molarity of 3 mM–80 mM by equimolar replacement of NaCl, CaCl_2_ was withdrawn and the resulting solution enriched with 0.1 mM of EDTA.

The procedure in these mechanism of action experiments entailed an initial recording of the normal spontaneous contraction of respective jejunal strip for 3 min’ prior addition of receptor blockers and enzyme inhibitors. The jejunal segments were then incubated with the respective receptor blockers for 2 min. The incubation period was also 2 min for methylene blue but 20 min for L-NAME as described by Li et al. (2018). Tracings of 3 min were recorded after incubation with receptor/enzyme blocker then followed by a 10-min recording upon addition of the respective TFG concentrations.

### Phytochemical Analysis of *T. fassoglense* Extract by LC-MS

Phytochemical analysis was performed via liquid chromatography-mass spectrometry (LC-MS). One Gram (1 g) of *T. fassoglense* extract was reconstituted in LC-MS water (1 mg/1 ml) and 20 μL injected in a quaternary LC pump (Model 1200) ESI-MS system coupled to Agilent MSD 6120-Triple quadruple MS with electrospray source (Palo Alto, CA). The sample were separated on a reverse-phase liquid chromatography on Agilent technologies 1200 infinite series, Zorbax SB C18 analytical column (2.1 mm x 50 mm, 1.8 um). The mobile phase consisted of water (eluent A) and acetonitrile (eluent B) for elution using a linear gradient program of: 0 min, 5% B; 0–5 min, 5–50% B; 5–10 min, 50–80% B; 10–15 min, 80–100% B; 15–25 min 100% B; 25–30 min 5% B; 30–35 min 5% B. Flow rate was held constant at 1 ml/min and the injection volume was 1.0 uL and the data was acquired in full-scan negative at 100–1500 m/z scan. The dwell time for each ion was 50 ms. Operating conditions were as follow for MS detection: capillary voltage, 3.0 kV; cone voltage, 70 V; extract voltage 5 V; radio frequency (RF) voltage, 0.5 V; source temperature, 110° C; nitrogen gas temperature for desolvation, 380° C; nitrogen gas flow for desolvation, 400 L/h. Linear calibration equation curve of peak area vs. concentration using following equation (*y* = 6008.9x-5250.3 (R2 = 0.9987) was used for external quantitation of all peaks.

### Ethical Considerations

Ethical approval to perform the study was sought from and given by Biosafety, Animal Use, and Ethics Committee in the Department of Veterinary physiology, the University of Nairobi (permit number RVM BAUEC/2020/260). All the protocols used in this study were in adherence with the National Institute of Health Guidelines for care and use of laboratory animals (8^th^ Edition), and obeyed the 4Rs (reduction, replacement, refinement, and rehabilitation) tenets of ethical experimental design.

### Statistical Analysis

The data obtained from the *in-vivo* and *ex-vivo* studies were expressed as mean ± standard error of mean (SEM) and analyzed using one-way ANOVA followed by Turkey’s post hoc test in cases of significance which was set at *p* < 0.05. Analysis was performed using GraphPad Prism^TM^ suite of statistical software.

## Results

### **Castor Oil-Induced Diarr**h**ea and Enteropooling Test**

The result of this experiment are shown in Table 1. There were significant differences both in fecal mass output and number of wet feces (*p* < 0.0001) in experimental groups. Post-hoc statistical analysis using Tukey’s multiple comparisons test revealed significant differences (*p* < 0.01) between the negative control and low dose test, medium dose test, high dose test, and positive control group. TFG extract not only demonstrated a dose-dependent effect on fecal mass output but also in the number of wet stools excreted by animal in the experimental groups within 4 h. The ED50 was 310 mg/kg.

**TABLE 1:** Effect of different doses of *Tylosema fassoglense* (TFG) in castor oil-induced diarrhea.

Fecal Mass and number (no.) of wet feces												
**Study groups**	0–1 h		1∼2 h		2∼3 h		3∼4 h		Total		Inhibition (%)	
	Fecal Mass	No. of wet Feces	Fecal Mass	No. of wet Feces	Fecal Mass	No. of wet Feces	Fecal Mass	No. of wet Feces	Fecal mass	No. of wet Feces	Fecal Mass	No. of wet Feces
Vehicle group	3.036 ± 1.29	4.0	2.070 ± 0.26	3.6	3.166 ± 1.18	3.8	1.77 ± 0.20	3.0	10.04 ± 0.71	14.4 ± 0.24		
TFG 200 mg/kg	2.54 ± 0.60	2.6	1.616 ± 0.38	2.0	0.432 ± 0.06*	1.8	0.658 ± 0.02	2.0	5.250 ± 0.46**	8.4 ± 0.51**	47.71	41.67
TFG 400 mg/ kg	0.800 ± 0.62	1.2	0.504 ± 0.33**	1.4	1.614 ± 0.71	1.4	2.234 ± 0.93	1.6	5.152 ± 0.58**	5.6 ± 0.24**	48.69	61.11
TFG 800 mg/kg	0.146 ± 0.06	0.6	0.244 ± 0.07**	0.8	0.814 ± 0.33	1.0	2.290 ± 0.18	2.0	3.494 ± 1.23**	4.4 ± 0.68**	65.20	69.44
Loperamide 5 mg/ml	0 ± 0*	0	0 ± 0**	0	0 ± 0**	0	1.182 ± 0.14	1.0	1.182 ± 0.26**	1.0 ± 0**	88.22	93.05

Results are expressed as mean ± SEM of 5 Sprague Dawley rats. *p<0.05, **p<0.01 compared with negative control. ANOVA followed by Turkey’s multiple comparison test.

The result of castor-oil induced enteropooling test is shown in Table 2. There were significant differences (*p* = 0.0004) between experimental groups in the mass of luminal content. Post-hoc statistical analysis using Tukey’s multiple comparisons test revealed significant differences (*p* < 0.05) between the negative control and the high dose test and positive control groups.

**TABLE 2:** The effects of different dosages of freeze-dried extract of *Tylosema fassoglense* on castor oil-induced enteropooling.

Enteropooling		
	Luminal content (g)	Inhibition (%)
Vehicle control	3.798	
TFG 400 mg/kg	3.236	14.80
TFG 800 mg/kg	2.820	25.75*
Positive control	2.160	43.13*

Results are expressed as mean ± SEM of five Sprague Dawley Rats. * p < 0.05 compared with negative control. ANOVA followed by Turkey’s multiple comparison test.

### Phenol Red Meal Transit Test

#### Gastric Emptying

The result of gastric emptying rate experiment is shown in Table 3. There were significant differences (*p* < 0.0001) in gastric emptying rates between experimental groups. Post-hoc statistical analysis using Tukey’s multiple comparisons test revealed significant differences (*p* < 0.01) between the negative control and high dose test group as well as between negative control and positive control groups.

**TABLE 3:** Effect of different *Tylosema fassoglense* (TFG) doses on gastric emptying rate in neostigmine-induced gastrointestinal motility.

Study groups	Dose (mg/kg)	Time before assaying for spectrophotometry (minutes)	Absorbency of gastric phenol red at 560 nm	Gastric emptying
Negative control	10	20	0.331	85.95 ± 3.39%
TFG	400	20	0.377	84.02 ± 0.65%
	800	20	1.121	52.47 ± 7.76%**
Loperamide	5	20	1.292	45.22 ± 3.98%**
Standard reference		0	2.509	0

Results are expressed as mean ± SEM of five Sprague Dawley Rats. **p < 0.01 compared with negative control. ANOVA followed by Turkey’s multiple comparison test.

#### Intestinal Transit Test

The results of intestinal motility experiment are shown inn Table 4. There were significant differences (*p* < 0.0001) in peristaltic indices between the experimental groups. Post-hoc statistical analysis using Tukey’s multiple comparisons test revealed significant differences (*p* < 0.0001) between the negative control and medium dose test, negative control and high dose test, as well as between the negative control and positive control groups.

**TABLE 4:** Effect of different *Tylosema fassoglense* (TFG) doses on intestinal transit rate in neostigmine-induced gastrointestinal motility.

Study groups	Dose(mg/kg)	Mean		
		Small intestine length (cm)	Phenol red meal distance traveled (cm)	% Intestinal transit
Negative control	10	125.38	104.54	83.37
TFG	400	119.44	71.46	59.82**
	800	120.54	67.08	55.64**
Loperamide	5	130.32	59.40	45.58**

Results are expressed as mean ± SEM of five Sprague Dawley Rats. **p < 0.01 compared with negative control.

### Isolated Rabbits Jejunum

#### Effect of Freeze-Dried Extract of TFG on Spontaneous and Acetylcholine-Induced Contraction

Various concentrations of the extract (0.5,1.0, 1.5, 2.0, 2.5, 3.0, 3.5 mg/ml) dose-dependently exhibited inhibitory effect on spontaneous contraction of isolate rabbit’s jejunal segments with an EC50 of 1.09 mg/ml. There were significant differences (*p* < 0.0001) in percentage inhibition of spontaneous jejunal force of contraction between different concentrations of the extract administered. Post-hoc statistical analysis using Tukey’s multiple comparisons test revealed significant differences between spasmolytic effect of 0.5 mg/ml and those exhibited by 1.0, 1.5, 2.0, 2.5, 3.0 and 3.5 mg/ml TFG also between the inhibitory effect of 1.0 mg/ml TFG extract and those displayed in 2.0, 2.5, 3.0, and 3.5 mg/ml TFG as well as between percentage contractions in presence of 1.5 mg/ml and those in presence of 3.5 mg/ml TFG. These results are shown in Table 5.

**TABLE 5:** Effect of different concentration of freeze dried extracts of *Tylosema fassoglense* (TFG) on spontaneous and acetylcholine-induced contraction of isolated rabbit’s jejunum.

% Contraction Inhibition		
**TFG dose ( mg/ml)**	Spontaneous contraction	Acetylcholine-induced contraction
0.5	8.44 ± 4.45^A^	7.19 ± 3.464^A^
1.0	35.15 ± 5.93^B^	39.48 ± 5.56^AB^
1.5	45.98 ± 4.28^BC^	67.56 ± 11.57^B^
2.0	59.17 ± 6.63^CD^	80.42 ± 6.661^C^
2.5	58.19 ± 5.82^CD^	75.71 ± 7.526^C^
3.0	68.01 ± 5.34^CD^	70.86 ± 10.66^BC^
3.5	74.80 ± 2.11^D^	74.44 ± 6.587^BC^

Results are expressed as mean ± SEM of five studies. Percentage mean contraction within each column that do not share similar letters denote statistical significance, p < 0.05. ANOVA followed by Turkeys multiple comparison test.

TFG in concentrations of (0.5,1.0, 1.5, 2.0, 2.5, 3.0, 3.5 mg/ml) dose-dependently attenuated the acetylcholine-induced contractions of the isolated rabbit’s jejunal segments with an EC50 of 0.83 mg/ml. There were significant differences (*p* < 0.0001) in percentage inhibition of acetylcholine-induced jejunal force of contraction exhibited by different extracts of TFG. Post-hoc statistical analysis using Tukey’s multiple comparisons test revealed significant differences (*p* < 0.05–0.01) between percentage contraction in presence of 0.5 mg/ml TFG and those exhibited in presence of 1.5, 2.0, 2.5, and 3.5 mg/ml TFG as well as between percentage contraction in presence of 1.0 mg/ml TFG and those in presence of 2.0, 2.5 mg/ml TFG as shown in Table 5.

#### Effect of Freeze-Dried Extract of TFG on 80 mM KCl and CaCl_2_-Induced Contraction

The isolated rabbit’s jejunal segments pre-contracted by 80 mMKCl in absence and presence of freeze dried TFG extract (0.5, 2.5, 3.5 mg/ml) exhibited significant differences (*p* < 0.0001) in percentage contractions. Post-hoc statistical analysis using Tukey’s multiple comparison test revealed significant differences (*p* < 0.01–0.05) between 100 ± 0.00% (negative control) vs. 53.14 ± 10.25% (2.5 mg/ml TFG) vs. 38.04 ± 4.712% (3.5 mg/ml TFG) as well as between 82.81 ± 7.48% (0.5 mg/ml TFG) vs. 53.14 ± 10.25% (2.5 mg/ml TFG) vs. 38.04 ± 4.712% (3.5 mg/ml TFG).

**FIGURE 1:**
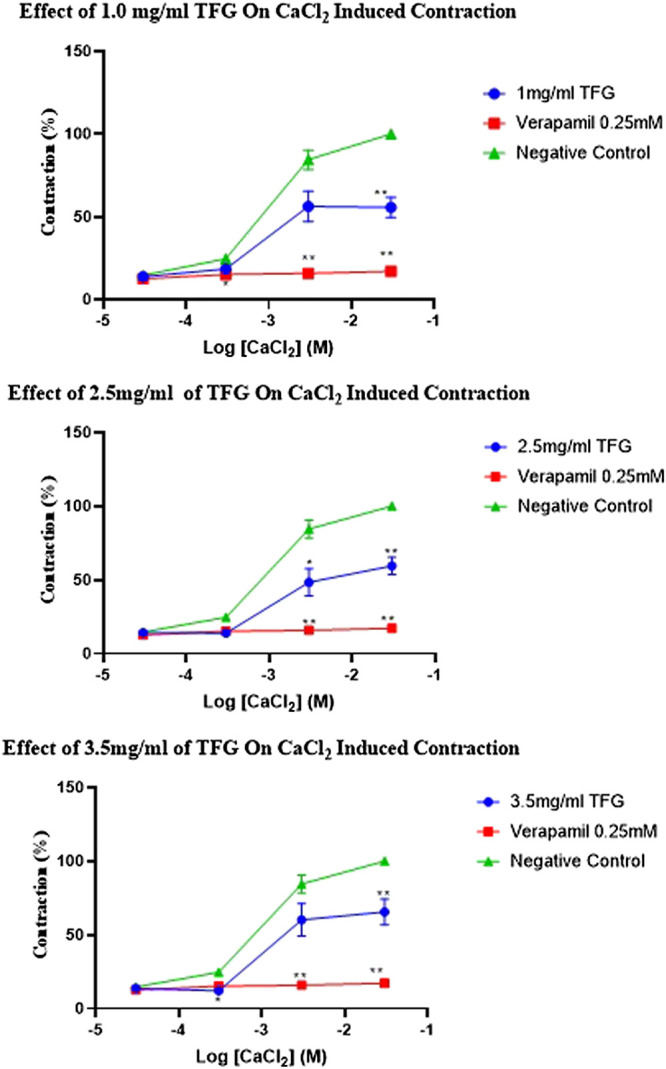
Effect of various doses of TFG and verapamil on CaCl_2_ induced contraction of an isolated rabbit jejunum. Compared with negative control group: *p < 0.05, **p < 0.01. ANOVA followed by Turkey’s multiple comparison test.

The percentage contraction caused by calcium chloride (0.00003–0.03 M) in absence and presence of freeze dried extract of TFG (0.5, 2.5, 3.5 mg/ml) and verapamil (0.025 mM) is as shown in Table 6 and Figure 1. There were no significant differences (*p* > 0.05) in percentage contraction caused by 0.00003 M calcium chloride both in presence and absence of TFG and verapamil.

**TABLE 6:** Effect of freeze dried extracts of various concentration of *Tylosema fassoglense* extracts and Verapamil on Calcium Chloride induced contraction on isolated rabbit’s jejunum.

% Contraction Inhibition				
**Treatment**	0.00003 M CaCl_2_	0.0003 M CaCl_2_	0.003 M CaCl_2_	0.03 M CaCl_2_
Negative control	14.65 ± 1.65	24.61 ± 2.01	84.49 ± 2.01	100 ± 0.00
TFG (mg/ml) 1.0	13.81 ± 1.84	18.45 ± 2.86	56.30± 9.28	55.62 ± 6.20*
2.5	14.18 ± 1.75	13.89 ± 2.22*	48.30 ± 9.36*	59.48 ± 9.36**
3.5	13.69 ± 1.13	12.02 ± 0.9*	60.18 ± 11.02	65.51 ± 8.78**
0.025 mM verapamil	12.56 ± 0.88	15.20 ± 2.12*	15.88 ± 2.39**	17.11 ± 2.45**

Compared with negative control group: *p < 0.05, **p < 0.01. ANOVA followed by Turkey’s multiple comparison test.

In presence and absence of freeze dried extracts of TFG and verapamil, 0.0003 M CaCl_2_ caused significant differences (*p* < 0.05) in percentage contractions. Post-hoc statistical analysis using Tukey’s multiple comparisons test revealed significant differences (*p* < 0.05) in jejunal contraction between negative control and in presence of 2.5 mg/m TFG, 3.5 mg/ml TFG, and verapamil.

Similarly, in absence and presence of freeze dried extract TFG and verapamil, 0.003 M CaCl_2_ caused significant differences in percentage contractions. Post-hoc statistical analysis using Tukey’s multiple comparisons test revealed significant differences (*p* < 0.05–0.01) in jejunal contraction between negative control and in presence of 2.5 mg/ml, 3.5 mg/ml TFG, and verapamil.

There were significant differences (*p* < 0.0001) in percentage contraction caused by 0.03 M CaCl_2_ in absence and presence of various concentration of TFG extract and verapamil. Post-hoc statistical analysis using Tukey’s multiple comparisons test revealed significant differences (*p* < 0.05–0.01) in jejunal contraction between negative control and in presence of 1.0 mg/ml, 2.5 mg/ml, 3.5 mg/ml TFG, and verapamil.

#### Effect of Various Blockers on TFG-Mediated Relaxation of Isolated Rabbit’s Jejunum

The percentage contraction inhibitions caused by 0.5 mg/ml TFG extract in absence and presence of various antagonist-pretreated jejunal segments (naloxone, methylene blue, L-NAME, prazosin, and propranolol) had no significant differences (*p* > 0.05).

There were significant differences (*p* = 0.0029) in percentage contraction inhibition caused by 1.0 mg/ml of freeze-dried extracts of TFG in absence and presence of various standard antagonists (naloxone, methylene blue, L-NAME, prazosin, and propranolol). Post hoc statistical analysis using Tukey's multiple comparisons test revealed significant differences (*p* = 0.0341) in percentage contraction between negative control and L-NAME pretreated jejunal segments.

TFG extract (1.5 mg/ml) exhibited significant differences (*p* < 0.0001) in percentage contraction inhibition in absence and presence of various standard antagonist (naloxone, methylene blue, L-NAME, prazosin, propranolol). Post hoc statistical analysis using Tukey's multiple comparisons test revealed significant differences (*p* < 0.001) in percentage contraction inhibition between negative control and those of L-NAME and methylene blue pretreated jejunal tissues.

There were significant differences (*p* = 0.0001) in percentage jejunal contraction inhibition caused by 2.5 mg/ml of freeze-dried extracts of TFG in absence and presence of various standard antagonists (naloxone, methylene blue, L-NAME, prazosin, and propranolol). Post hoc statistical analysis using Tukey's multiple comparisons test revealed significant differences (*p* < 0.001) in percentage contraction inhibition between negative control and contractions in jejunal segments pretreated with L-NAME, methylene blue, and naloxone.

**FIGURE 2:**
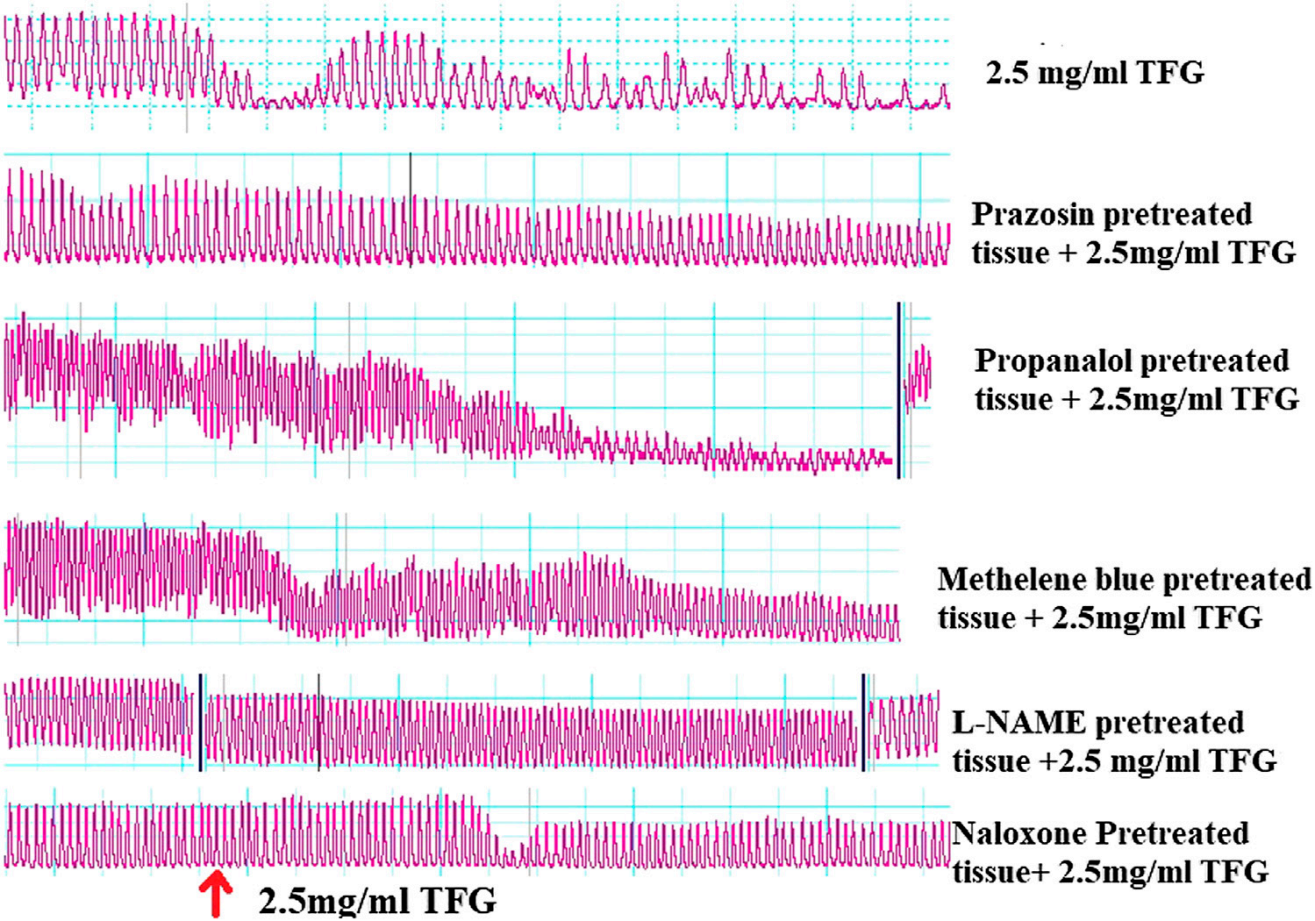
Representative Powerlab tracing showing the effect of freeze-dried extract of TFG on spontaneous contraction, prazosin, propranolol, methylene blue, L-NAME and naloxone pretreated tissue.

TFG extract with concentration strength of 3.5 mg/ml caused significant differences (*p* = 0.0014) in percentage contraction inhibition in absence and presence of standard antagonists (naloxone, methylene blue, L-NAME, prazosin, and propranolol). Post hoc statistical analysis using Tukey's multiple comparisons test revealed significant differences (*p* < 0.05) in percentage contraction inhibition between negative control and L-NAME and methylene blue pretreated jejunal segments. The tabulation of these results and representative Powerlab tracing are shown in Table 7 and Figure 2 respectively.

**TABLE 7:** Effect of freeze dried extracts of TFG in presence of various antagonists (10^–4^ M) on *Tylosema fassoglense* (TFG) extract-mediated relaxation of isolated rabbit’s jejunum.

% Contraction Inhibition						
**TFG dose (mg/ml)**	Negative control	Naloxone	Methylene blue	L-NAME	Prazosin	Propranolol
0.5	8.44 ± 4.45	12.35 ± 4.17	6.29 ± 2.14	2.52 ± 1.23*	7.30 ± 4.09	23.64 ± 5.25
1.0	35.15 ± 5.93	42.27 ± 9.92	7.31 ± 8.92*	2.09 ± 1.23**	27.64 ± 9.048	29.82 ± 3.33
1.5	45.98 ± 4.28	22.10 ± 3.44	11.03 ± 4.66**	8.78 ± 7.20**	39.50 ± 6.97	45.32 ± 3.69
2.5	59.17 ± 6.63	25.81 ± 3.84*	24.70 ± 6.29**	14.15 ± 2.63**	57.26 ± 5.35	55.22 ± 2.59
3.5	74.80 ± 2.11	40.73 ± 5.00	25.50 ± 9.34**	28.67 ± 12.44**	58.90 ± 2.32	57.55 ± 10.87

Results are expressed as mean ± SEM of five studies. *P < 0.05, **p < 0.01 compared with negative control in each TFG concentration. ANOVA followed by Turkeys multiple comparison test.

### Phytochemical Profile of *Tylosema fassoglense*

**FIGURE 3:**
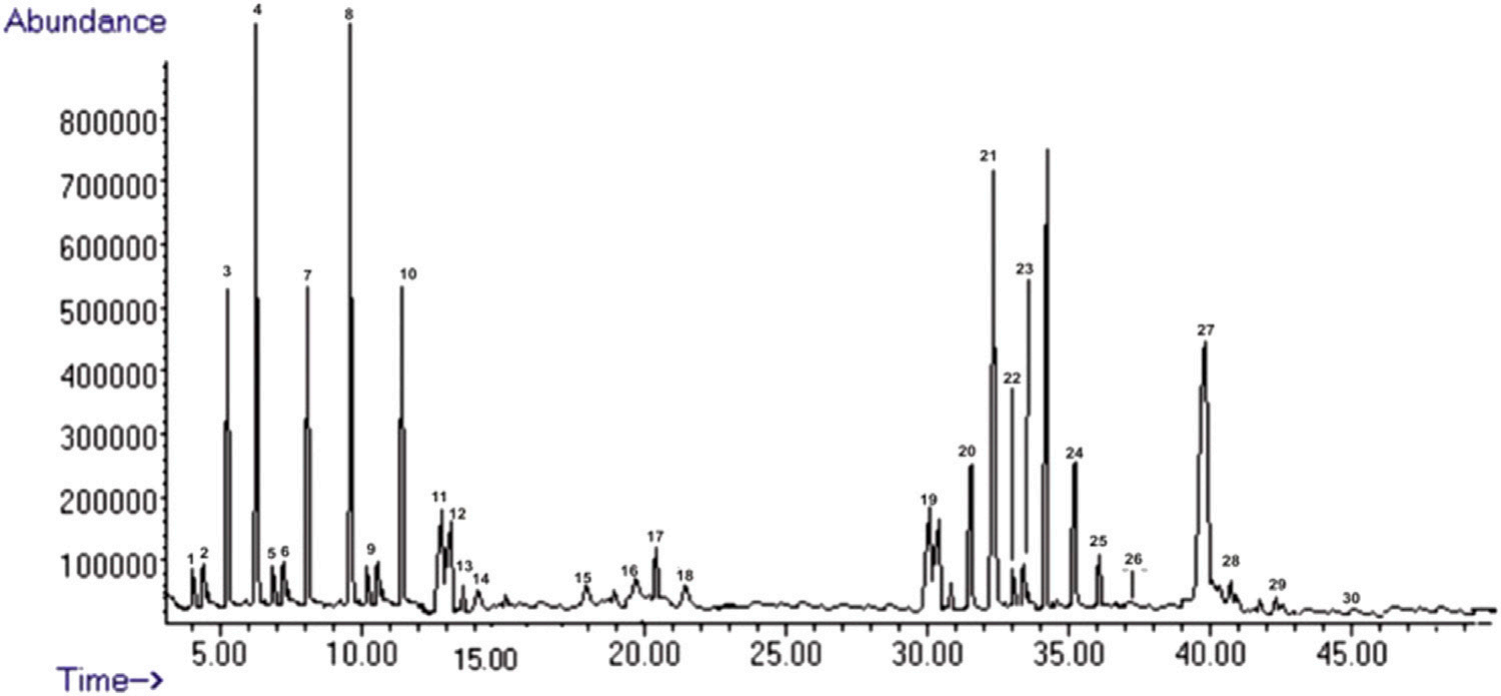
The LC-MS chromatograms of freeze-dried extract of *Tylosema fassoglense*.

Chemical constituents in freeze-dried extracts of *Tylosema fassoglense* were characterized using LC-MS method where a total of 30 compounds were identified and characterized as shown in Table 8: twelve phenolic compounds (1–11, 16), fourteen flavonoids (12–15, 17–24, 27–28), and four alkaloids (25–26, 29–30). The chromatogram is shown in Figure 3. These 30 identified compounds represented the major components in freeze dried extracts of *Tylosema fassoglense*.

**TABLE 8:** *Tylosema fassoglense* profile determined by LC-MS.

Number	Rt	Name	Ion mode	(M-H)	% Abundance
1	4.79	Gallic acid	Neg	169.1	1.2734
2	5.02	Protocatechuic acid	Neg	353	2.0882
3	6.33	4-Hydroxybenzoic acid	Neg	153	6.1958
4	7.03	Syringic acid	Neg	183	7.3075
5	7.41	Caffeic acid	Neg	179	1.0076
6	7.88	P-Coumaric acid	Neg	151	0.9068
7	8.92	Sinapic acid	Neg	163	4.2437
8	9.97	M-Coumaric acid	Neg	164	3.4253
9	12.9	Ferulic acid	Neg	609	1.7985
10	13.4	Cinnamic acid	Neg	611	5.1324
11	14.2	Catechin	Neg	463.1	3.1143
12	14.3	Rutin	Neg	137	2.2965
13	14.4	Naringin	Neg	138	0.7547
14	14.5	Hesperidin	Neg	317	0.5197
15	18.7	Fisetin	Neg	285	1.5329
16	22.3	Coumarin	Neg	147	1.3976
17	22.9	Quercetin	Neg	300.9	2.1639
18	23.2	Naringenin	Neg	271	1.1987
19	31.4	Hesperitin	Neg	301	3.9876
20	33.5	Luteolin	Neg	285	4.0843
21	33.7	Kaempferol	Neg	285.2	3.5478
22	33.8	Epigenin	Neg	269	2.6432
23	34.2	Rhamnetin	Neg	315.3	1.5435
24	37.5	Chrysin	Neg	253	2.5423
25	38.02	Chelerythine	Neg	537.2	3.6432
26	38.4	Piperine	Neg	653	1.5423
27	40.2	Proathocyanidin	Neg	753	11.5423
28	43.3	Quercitanin	Neg	553	1.5423
29	43.8	Hygrine	Neg	453	0.5423
30	45.1	Coniine	Neg	372	0.2423

## Discussion

This study aimed to investigate the antidiarrheal activity of aqueous extract of tubers of *Tylosema fassoglense* in both *in-vivo* and *ex-vivo* animal models.

The freeze-dried extracts of TFG caused significant reductions in fecal mass, number of wet stool excreted, and luminal enteropooling in the castor oil model of diarrhea. All the three doses of the extract tested had a significant effect on fecal mass and number of wet feces excreted but only the high dose of TFG (800 mg/kg) had a significant effect on enteropooling which is an indication that the extract possess antidiarrheal activity. The orally administered castor oil undergoes digestion by pancreatic lipase in the small intestine resulting in the liberation of ricinoleic acid, a biologically active molecule (Awouters et al., 1978) which mediates its effects via activation of G-protein coupled E3 and E4 prostanoid receptors (Tunaru et al., 2012). The E3 Prostanoid receptors are predominantly expressed in intestinal and uterine smooth muscle (Bos et al., 2004). Ricinoleic acid binds on the prostanoid receptor E3 whose downstream signaling results in a reduction in the cytosolic cAMP concentration via G_i -_mediated inactivation of adenylyl cyclase (Bos et al., 2004). High concentrations of cAMP in the cytosol has been shown to cause calcium desensitization (Fukata et al., 2001) and thus its reduction has an eventual effect of increased force of contraction of the intestinal smooth muscle.

Ricinoleic acid has also been shown to bind onto the prostanoid receptor E4 that is predominantly distributed on the intestinal epithelial mucosa (Bos et al., 2004). Activation these receptors lead to elevation of cAMP via sequential activation of Gs and adenylyl cyclase which causes chloride hypersecretion through the cAMP-gated cystic fibrotic regulator (CFTR) channels with subsequent luminal fluid shift (Bos et al., 2004). The accumulating luminal fluids provide an additional propulsive drive that shorten the transit time leading to diarrhea (Awouters et al., 1978). The extract’s significant effect in reducing bowel movement in all the doses tested but exhibiting antisecretory activity only at the highest dose tested in the study is probably because it mediates its effect mainly via blockade of the E3 prostanoid receptors due to their greater levels of expression.

The freeze-dried extracts of TFG possessed significant effects on the peristaltic index at all the doses tested but only exhibited a significant effect on gastric emptying rate at the highest doses tested in the neostigmine-induced gastrointestinal motility experiment. Neostigmine is an indirect cholinergic agonist that inhibits the activity of acetylcholinesterase enzyme thus elevating the levels of endogenous acetylcholine to a mild cholinergic toxidrome at a dose of 0.1–0.05 mg/kg (Li et al., 2018). The observed overall effect is as result of an increased enteric cholinergic activity on muscarinic receptors where activation of M2 receptors lowers cytosolic cAMP levels and thus increased calcium sensitization while M3 activation increases cytosolic calcium ions (Li et al., 2018).The net effect is increased phosphorylation of myosin light chain and subsequent increased force of contraction leading to increased gastrointestinal motility (Fukata et al., 2001). Rapid gastric emptying has been shown to amplify intestinal motility which causes diarrhea (Li et al., 2018).

It is noteworthy that the extract caused significant reduction in intestinal transit rate of phenol red meal in all doses tested but only the highest dose had significant effect on gastric emptying rate. The most likely explanation for this seemingly contradictory observation is anchored on the premise that the physiological forces governing gastric emptying and intestinal motility are qualitatively different (Dooley et al., 1984). The stomach consists of two physiological divisions; the reservoir (composed of cardia, fundus, and upper one third of corpus) with the properties of receptive relaxation and low motility. The second is the antral pump (composed of lower two thirds of corpus, antrum, and the pyloric sphincter) which possesses retropulsive motility patterns (Dooley et al., 1984). It has also been shown that stomach has complex involvement of neural and hormonal signals during gastric emptying (Gondim et al., 1998; Li et al., 2018). Unlike stomach, the small intestines have relatively uniform motility pattern. It is clear from the foregoing discussion that the stomach would be expected to have a more complex response to pharmacological stimulation compared to intestines and this forms a plausible explanation for the difference in response to the extract. The observed antimotility effect are probably mediated via anticholinergic action of the phytochemical components of the extracts.

*Ex-vivo* studies using isolated rabbit’s jejunum were carried out to investigate the dose dependency of spasmolytic effect as well as the putative mechanisms of action of these spasmolytic effects.

The freeze-dried extract of TFG had significant concentration-dependent i.e., dose-dependent inhibition of the spontaneous contraction of the isolated jejunal segments. The *in-vitro* spasmolytic effects of TFG are an indication that the phytochemical constituents are intrinsically active and do not necessarily need microbiotic or metabolic biotransformation into active molecules to exert their actions.

The freeze-dried extract of TFG had significant concentration-dependent inhibition of acetylcholine-induced contraction of the isolated jejunal segment. This demonstrates the possible presence of anticholinergic compounds within the extract a finding that correlates with the findings in the *in-vivo* studies where TFG significantly reduced gastrointestinal motility induced by neostigmine. The extracts may also possess alternative compounds that cause blockade of influx of Ca^2+^ ions that are mandatory for the effective smooth muscle contraction (Jiang and Stephens, 1994).

The extract significantly inhibited the spasmogenic effect of 80 mM KCl induced contraction of isolated jejunal segments. Substances with spasmolytic effect on smooth muscle contraction induced by high potassium chloride concentration solutions (KCl > 30 mM) are considered calcium channel blockers (Vogalis, 2000; Li et al., 2018). This is because high potassium chloride concentration solutions cause membrane depolarization with the subsequent opening of voltage gated calcium channels as demonstrated in the patch-clamp studies (Vogalis, 2000). This results in an unregulated influx of calcium ions and thus the observed tonic contraction. Therefore, the spasmolytic effect of TFG on high potassium chloride induced contraction is indicative of a possible blockade of voltage gated calcium channels.

The TFG extract had significant effects on calcium chloride-induced contraction and the effects (as shown by concentration-response curves) paralleled those of verapamil, a use-dependent dihydropyridine channel blocker (Vogalis, 2000). The observed effect of TFG on calcium chloride-induced contraction is therefore confirmatory of extract’s blocking activity on voltage-gated calcium channels.

L-NAME and methylene blue significantly and independently attenuated the spasmolytic activity of the freeze-dried extracts of TFG. L-NAME is a nitric oxide synthase inhibitor. Nitric oxide synthase catalyzes the cleavage of endothelial L-arginine into citrulline with the liberation of nitric oxide gas in an oxygen and NADPH dependent reaction (Cartledge et al., 2001). Methylene blue on the other hand inhibits soluble guanylyl cyclase (Ragy and Elbassuoni, 2012). Nitric oxide (NO) activates soluble guanylate cyclase by binding on its heme component (Cartledge et al., 2001). Guanylyl cyclase hydrolyzes GTP into cyclic GMP which activates protein kinase G (PKG). PKG phosphorylates serine and threonine amino acid residues of telokin, a segment of myosin light chain kinase (MLCK), without affecting the kinase and calmodulin binding domains, thereby reducing activity of MLCK (Khromov et al., 2006). This enhances the activity of myosin phosphatase that cause calcium desensitization (Khromov et al., 2006). This ultimate effect is smooth muscle relaxation (Sanders et al., 2012). The observed attenuation of the extract’s spasmolytic effect in L-NAME and methylene blue pre-treated jejunal segments indicates that the spasmolytic effects of TFG extracts may be partially mediated by modulation of the NO-cGMP pathway.

Prazosin and propanol pretreated tissues did not possess significant effect on freeze-dried extracts of TFG-mediated relaxation of the isolated jejunal segments. This indicates that the spasmolytic activities of the freeze-dried extracts of TFG are probably not mediated via modulation of adrenergic receptors.

The *ex-vivo* findings indicates that the TFG extract possibly mediates its antidiarrheal effect via modulation of the NO-cGMP pathway, blockade of voltage gated calcium channels, and muscarinic receptor blockade. These pharmacological effects result in smooth muscle relaxation and a consequent increase in gastrointestinal transit time which enhances intestinal fluid absorption and thus reduces fecal mass. This in turn reduces frequency of bowel movement manifesting in overall antidiarrheal effect. The extracts displayed antisecretory activity at high doses which were possibly mediated by blockade of prostanoid EP4 receptors further modifying the volume and consistency of fecal matter.

The LC-MS phytochemical profile of TFG indicated presence of sizeable amounts of Proathocyanidin and Luteolin. Luteolin has been reported to possess significant *in-vivo* antidiarrheal effect in mice (Sadraei et al., 2019). Proathocyanidin on the other hand has been shown to activate the NO-sGC-cGMP pathway in *ex-vivo* studies using isolated aorta of a rat (DalBó et al., 2008). The presence of these substances may therefore partly explain the significant effect of TFG extracts observed in this study.

There are several possible explanations of the TFG’s multiple spasmolytic signaling pathways as seen in this study. The first likely reason is based on the premise that plant extracts are multicomponent mixtures of bioactive compounds, some of which may act synergistically or antagonistically. Indeed, The LC-MS characterized 30 compounds (phenolic, flavonoids, and alkaloids) which possibly act via different signaling pathways. Secondly, smooth muscles have a pharmacomechanical coupling property and its accepted that acetylcholine may mediate its actions on smooth muscle and neural tissue by modulating calcium flux via both VGCCs (Roberts-Crowley and Rittenhouse, 2018; Brown, 2019). This may partially explain these multiple pathways some which are seemingly contradictory to what is expected.

There are some potential limitations of this study. Firstly, due to limited resources, in this case the only available force transducer in the Langendorff organ bath is more sensitive to rabbit’s jejunal contractions than those of rat’s jejunum. Hence, the study utilized a mixed animal model design i.e., Sprague Dawley rats in the *in-vivo* and isolated rabbit’s jejunum in the *ex-vivo* studies. Therefore, the *in-vivo* results should be interpreted with caution alongside the *ex-vivo* findings since biological differences exists in animal species. Secondly, since the phenol red experiments were conducted under conditions where neostigmine had been administered to experimental animals, the question as to whether the extracts can cause gastroparesis remain unanswered. It is however our considered view that this question can answered in future studies. In addition, both acute and chronic toxicities studies on tubers should be carried. It should be noted however that no signs of toxicities were observed during conduct of this studies even at the highest doses used i.e. 800 mg/kg.

In conclusion, the aqueous tuber extracts of TFG possessed significant antidiarrheal effect in the *in-vivo* and spasmolytic effect in *ex-vivo* studies that are probably mediated via modulation of nitrous oxide, calcium channel, and muscarinic pathways which would seem to validate the traditional use of the plant as an antidiarrheal medicine. These putative pharmacological actions are an indication that these tubers would potentially be effective in multiple categories of diarrhea since they possess both antisecretory and spasmolytic activities. Future studies should aim at trying to determine which of the major chemical moieties found in the tubers are mainly responsible for mediating the antidiarrheal effects. In addition, although the results of this study indicate that extract derived from tubers of *Tylosema fassoglense* possess significant antidiarrheal activity, commercial exploitation of this species should only take place after establishment of commercial growing program so as to ensure sustainability.

## Data Availability

The raw data supporting the conclusions of this article will be made available by the authors, without undue reservation.

## Abbreviations

CFTR, cystic fibrotic transmembrane regulator; CMC, carboxy methyl cellulose; eNOS, endothelial nitric oxide synthase; EP-prostanoid receptors; LC-MS, liquid chromatography and mass spectroscopy; MLCK, myosin light chain kinase; MOR, mu opioid receptors; MP, myosin phosphatase; RA, Ricinoleic acid; sGC-soluble guanylyl cyclase; TFG, *Tylosema fassoglense*; VGCC, voltage gated calcium channels.
